# Distinct Pools of Non-Glycolytic Substrates Differentiate Brain Regions and Prime Region-Specific Responses of Mitochondria

**DOI:** 10.1371/journal.pone.0068831

**Published:** 2013-07-17

**Authors:** Do Yup Lee, Zhiyin Xun, Virginia Platt, Helen Budworth, Christie A. Canaria, Cynthia T. McMurray

**Affiliations:** 1 Life Sciences Division, Lawrence Berkeley National Laboratory, Berkeley, California, United States of America; 2 Department of Advanced Fermentation Fusion Science and Technology, Kookmin University, Seuol, Korea; 3 The Buck Institute for Research on Aging, Novato, California, United States of America; Mayo Clinic, United States of America

## Abstract

Many hereditary diseases are characterized by region-specific toxicity, despite the fact that disease-linked proteins are generally ubiquitously expressed. The underlying basis of the region-specific vulnerability remains enigmatic. Here, we evaluate the fundamental features of mitochondrial and glucose metabolism in synaptosomes from four brain regions in basal and stressed states. Although the brain has an absolute need for glucose *in vivo,* we find that synaptosomes prefer to respire on non-glycolytic substrates, even when glucose is present. Moreover, glucose is metabolized differently in each brain region, resulting in region-specific “signature” pools of non-glycolytic substrates. The use of non-glycolytic resources increases and dominates during energy crisis, and triggers a marked region-specific metabolic response. We envision that disease-linked proteins confer stress on all relevant brain cells, but region-specific susceptibility stems from metabolism of non-glycolytic substrates, which limits how and to what extent neurons respond to the stress.

## Introduction

Dysfunction of mitochondria (MT) is thought to be a primary contributor to aging and neurodegenerative disease, but its role is poorly understood [1], [2]. Most if not all patients with neurodegeneration share the property that MT are, in general, not keeping up with the energy demands of the cell [3], yet typically, only particular regions are initially targeted for death [4]. For example, in Alzheimer’s disease (AD), beta-amyloid toxicity is prominent in the hippocampus (HIP) [5], [6], while toxicity observed in spinocerebellar ataxia type I (SCA1) manifests in the cerebellum (CBL) [7], [8] and Huntington’s disease (HD) primarily targets the striatum (STR) [9], [10]. Region-specific cell death implies that mitochondrial dysfunction develops in response to a changing cellular metabolism [11], [12], but the metabolic basis for regional toxicity remains one of the most puzzling features of neurodegenerative disease.

Glucose utilization is a requirement for brain function [12]. Low glucose is a prominent feature of patients with neurodegeneration [13] and in animal models [14]. However, it is not obvious how a global suppression of glucose utilization accounts for the region-specific susceptibility to death. In the R6/2 HD mouse model, for example, glucose levels correlate directly with the cerebral blood volume (CBV) in regions of the brain, except in the affected STR and neocortex, where CBV is abnormally high [14]. Thus, affected brain regions in this model have equivalent or greater access to glucose relative to regions more resistant to toxicity. The mismatch between CBV and low glucose in R6/2 animals does not correlate with the degree of atrophy, or the cell number in the affected regions [14]. Collectively, the results imply that region-specific neuronal toxicity reflects inherent differences in metabolism of glucose rather than its availability.

Defining inherent differences in glucose metabolism in disease states is challenging since basal metabolism is poorly understood and difficult to assess. Metabolites are in constant flux, and enter and leave a region of interest by multiple routes, making it difficult to quantitatively account for region-specific metabolism *in vivo.* Isotopic labeling provides insight into the processing of individual metabolites, but does not provide a global perspective [15] in which substrates and products are generated and consumed simultaneously as an integrated unit. In general, whole-organ measurements using 2-deoxyglucose (2-DG) and positron emission tomography (PET) provide powerful means to determine the glucose requirements in each brain region [16]–[18]. However, these measures do not reveal how glucose is processed in distinct brain regions, or the basis for heterogeneous glucose utilization among brain regions.

To address these issues, we have evaluated metabolism in four regions of a normal mouse brain in the resting and stressed states. We used mass spectrometry to develop a map of region-specific metabolites, and a statistical approach that allows for single and simultaneous comparison among all four brain regions on a global scale. We integrated the map with the synaptosomal bioenergetics, and tested the underlying basis for region-specific differences in glucose metabolism. Although the brain has an absolute need for glucose, we report here that brain synaptosomes are not strongly glycolytic. Rather, a remarkably distinct “signature” pool of non-glycolytic substrates characterizes each brain region, and is used during energy crisis. We envision that non-glycolytic substrates equip each region with a unique capability for energy production, that is, at least partially, independent of exogenous glucose availability. Use of non-glycolytic substrates provides a basis for differential glucose utilization and region-specific responses to stress.

## Results

**Figure 1:**
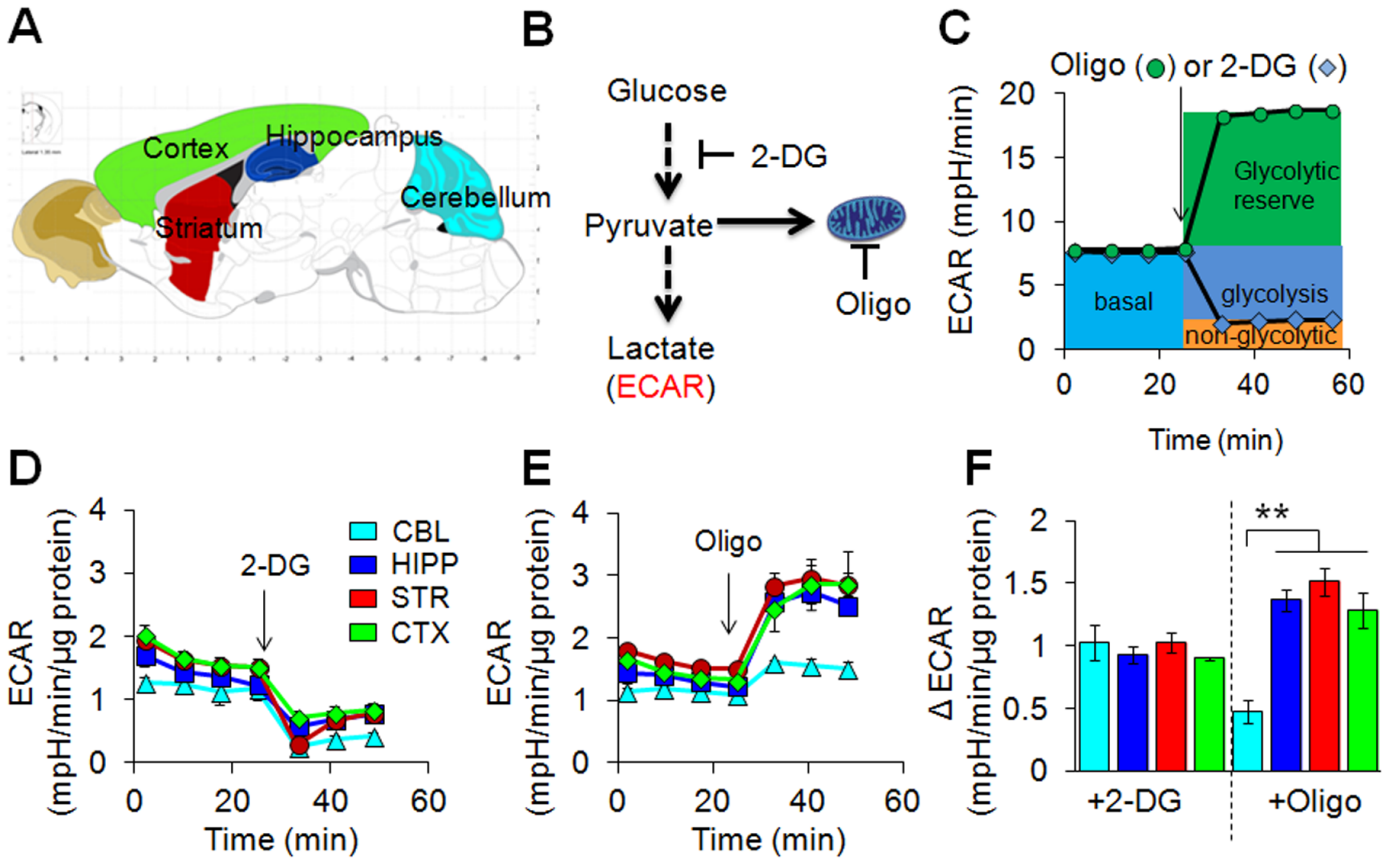
Synaptosomes from distinct brain regions have comparable capacity for glycolysis under basal conditions. (**A**) Schematic representation of the dissected regions of the brain for synaptosomal isolation. Cerebellum (CBL), hippocampus (HIP), striatum (STR), and cortex (CRT) are indicated by color. (**B**) A simplified pathway for glucose metabolism. Glucose is converted to pyruvate and pyruvate is converted to lactate, which is measured by the extracellular acidification rate (ECAR). (**C**) A schematic diagram defining the measurement of the glycolysis and glycolytic reserve by ECAR. 2-DG is an inhibitor of the glycolytic pathway (blue diamonds) and oligomycin (Oligo) an inhibitor of ATP synthase (green circles). ECAR decreases upon 2-DG addition and defines glycolytic activity. The remaining ECAR represents non-glycolytic acidification. The glycolytic reserve, which is drawn upon during the oligomycin block, is obtained by complete inhibition of mitochondrial respiration with oligo. (**D,E**) Representative glycolytic rates of brain regional ECAR under basal conditions and upon injections of 2-DG (**D**) and oligomycin (**E**). Each profile represents one independent biological experiment analyzed in triplicate. Data are means ± SEM (n = 3). The arrows indicate the injection of inhibitors. Three independent experiments were performed to obtain quantification of glycolytic parameters presented in (**F**). (**F**) Changes in ECAR after addition of 2-DG and oligo. Isolated synaptosomes from CBL, HIP, STR, and CRT exhibit low and comparable rates of glycolysis while the synaptosomes from CBL exhibit lower glycolytic reserve relative to HIP, STR, and CRT. Color key is indicated. Three independent experiments were performed, and each was performed with two mice that were analyzed in triplicate. Data are mean ± SEM (n = 3). **P<0.0008 with one-way ANOVA and Fisher’s LSD.

To measure differences in metabolism, we dissected four regions of the normal brain including cortex (CTX), STR, HIP, and CBL from C57BL/6 mice of 12–16 weeks (Figure 1A). Brain regions are complex and the number of neurons and glia vary dramatically among brain regions, ranging from 5-fold excess of neurons to 11-fold excess of glial cells [19]. Therefore, in this report, we evaluated the bioenergetics of isolated synaptosomes to determine whether there were intrinsic region-specific differences among neurons, and, in parallel, measured the metabolic content of each dissected region using gas chromatography-coupled mass spectrometry (GC-MS). Synaptosomes are “pinched off” nerve terminals that harbor intact neuronal MT within a physiological milieu [20]. The GC/MS provides a broad coverage of primary metabolites and captures the complex metabolic microenvironment to which the neurons respond. In all experiments, the dissected brain regions were isolated and measured simultaneously for robust comparison of their regional metabolic profiles and their bioenergetic differences.

### Synaptosomes from Distinct Brain Regions have Comparable Bioenergetics under Basal Conditions

Glycolysis and oxidative phosphorylation are the two major energy-producing pathways in the cell [12]. Thus, we measured whether there was differential usage of either bioenergetic pathway in synaptosomes from distinct regions of the brain [21]. In the first experiment, we supplied the synaptosomes with glucose as the exogenous substrate, added the glycolytic inhibitor, 2-DG [16]–[18], and tested whether glycolysis differed among synaptosomes in any of the brain regions. Glycolysis was inferred by the extracellular acidification rate (ECAR) when glucose is converted into lactic acid (Figure 1B) [22]. Because glycolysis dominates ECAR [23], we anticipated that it would decrease with 2-DG treatment (Figure 1C, glycolysis). Indeed, the addition of 2-DG resulted in comparable decrease of ECAR in all four regions of the brain (Figure 1D,F). As judged by GC/MS, there were no consistent intensity patterns among the glycolytic substrates or products, implying that the intermediates of the glycolytic pathway in each brain region were similar (Figure S1).

As a complementary approach, we supplied synaptosomes with glucose and measured ECAR after blocking oxidative phosphorylation with the addition of oligomycin (Oligo), an inhibitor of ATP synthase (Figure 1B,C) [24]. Oligomycin treatment leads to an increase in ECAR as the use of glycolysis increases to compensate for the loss of ATP generation from oxidation phosphorylation (Figure 1C, glycolytic reserve). ECAR doubled in the STR, HIP, and CTX (Figure 1E,F), and increased approximately 60% in the CBL (Figure 1E,F). Thus, with the exception of the CBL, glycolytic activity was similar in neuronal synaptosomes from each brain region under basal conditions.

**Figure 2:**
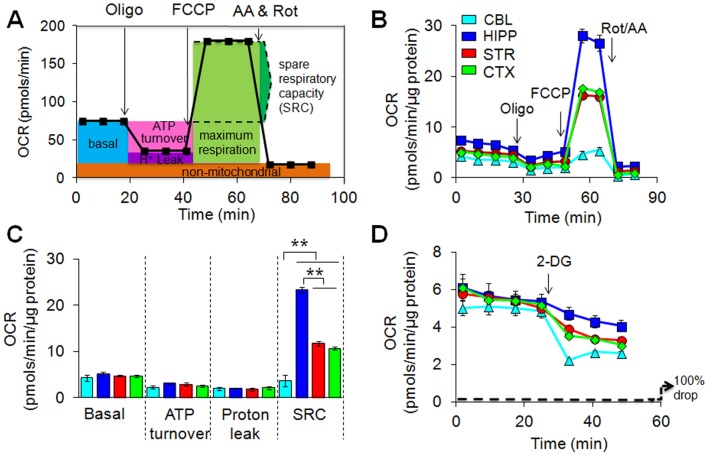
Oxidation of non-glycolytic substrates is prominent in brain synaptosomes in the basal state. (**A**) Schematic diagram delineating functional effects of electron transport chain inhibitors on mitochondrial respiration. A typical experiment involves measuring the OCR at the resting state (basal respiration) followed by injection of oligo (inhibitor of ATP synthase), and the drop in the OCR represents ATP turnover. Subsequent injection of FCCP dissipates the proton gradient and allows maximum respiration. The rise in OCR (relative to the basal respiration) upon FCCP addition represents mitochondrial spare respiratory capacity (SRC). Finally, a cocktail of rotenone (Rot) and antimycin A (AA) are added to completely disable the electron transport chain and inhibition of the total mitochondrial respiration. The remaining OCR after complete inhibition of mitochondrial respiration represents non-mitochondrial respiration. The OCR difference between oligo- and Rot and AA-responsive OCR reflects proton leak. (**B**) Representative profiles of brain regional OCR under basal conditions and upon injections of mitochondrial inhibitors. Each profile represents one independent biological experiment analyzed in triplicate. Data are means ± SEM (n = 3). The arrows indicate the injection of mitochondrial inhibitors. Three independent experiments were performed to obtain quantification of mitochondrial functional parameters presented in (**C**). (**C**) Quantification of mitochondrial functional parameters. Basal respiration, ATP turnover, and proton leak do not significantly differ among the four regions, but SRC is the highest in the HIP, comparably moderate in the STR and CTX, and the lowest in the CBL. Three independent experiments were performed. Data are means ± SEM (n = 3). **P<0.0003 with one-way ANOVA and Fisher’s LSD. (**D**) Representative profiles of brain regional OCR under basal conditions and upon injections of 2-DG. Each profile represents one independent biological experiment analyzed in triplicate. Data are means ± SEM (n = 3). The arrows indicate the injection of the glycolytic inhibitor 2-DG. The dotted line indicates 100% loss of OCR. Quantification of independent experiments is presented in Figure 6C.

Oxidative phosphorylation also did not distinguish regional synaptosomes when glucose was the exogenous substrate. Actively respiring MT consume oxygen as ADP is converted into ATP, and the oxygen consumption rate (OCR) correlates with the activity of the electron transport chain (Figure S2) [12], [23], [25]. However, in the presence of glucose, neither ATP turnover nor proton leak [25], [26](defined in Figure 2A and Figure S2) distinguished synaptosomes from the CBL, STR, CTX, and HIP (Figure 2B,C). Inhibiting ATP synthase with oligomycin resulted in a decrease in OCR, but the decrease was similar among regions (Figure 2B,C). As measured by GC/MS, there were no region-specific patterns for tricarboxylic acid (TCA) intermediates in the brain extracts (Figure S3). Thus, purified synaptosomes from all four regions were equally capable of substrate oxidation under basal conditions, and had comparable precursors to do so.

### The Majority of Mitochondrial Metabolism in Brain Synaptosomes Arises in the Basal State from Oxidation of Non-glycolytic Substrates

Synaptosomes in the four brain regions displayed similar glycolytic activity and mitochondrial respiration under basal conditions. To test whether glucose served as the primary energy source, we supplied synaptosomes with exogenous glucose, but blocked its utilization with 2-DG. Under these conditions, OCR depends entirely on endogenous non-glycolytic substrates. We anticipated that OCR would decrease, and the degree of the decrease would reflect the dependence on glycolysis. However, the decrease in OCR was unexpectedly modest in synaptosomes from all four brain regions (Figure 2D). OCR decreased to roughly half of its basal rate in the CBL, by only 30% in the STR and CTX, and we observed no significant reduction in the HIP (Figure 2D). The large residual OCR after the 2-DG block implied that most of the ATP production in the basal state arose from oxidation of non-glycolytic substrates. Although the OCR overall was similar among the STR, CTX and HIP, blocking with 2-DG differentiated the regions.

### MT from Functionally Defined Brain Regions have Signature Non-glycolytic Pools as Substrates for Energy Production

**Figure 3:**
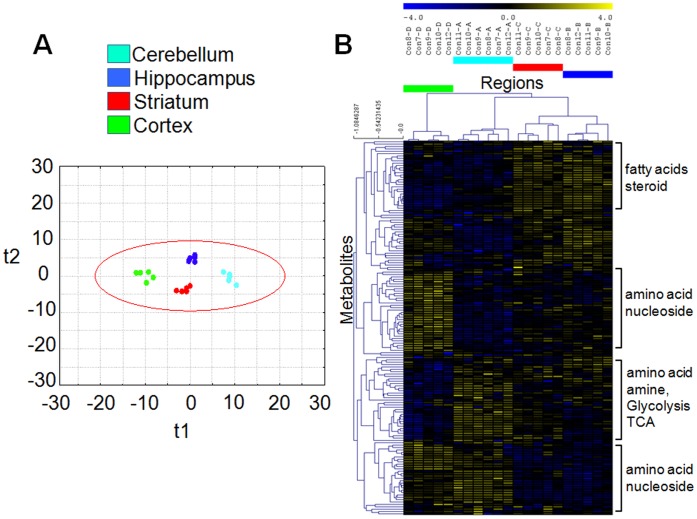
Partial least squared (PLS) statistics and clustering analysis of region-specific brain indicate differences in metabolomes. (**A**) PC 1 (t1) and PC 2 (t2) shows the separation of the metabolome among four different brain regions. PC 1 (30.5% total explained variance) discriminated metabolite profiles of STR and HIP from CRT and CBL. PC 2 (6.0% total explained variance) primarily separated clusters between STR and HIP (n = 5 or 6). Red circle indicates 3×S.E. (**B**) Hierarchical clustering analysis showing chemically/biochemically classifies metabolites clustered according to different brain regions (n = 5 or 6). The color code; CBL: teal, HIP: blue, STR: red, CTX: light green.

To test whether and what types of non-glycolytic metabolites might differentially contribute to region-specific metabolism, we extended our GC/MS profiling to identify a broad range of primary metabolites in the four regions of the normal brain (Table S1 in File S1). Indeed, region-specific differences in non-glycolytic substrates were evident by multivariate statistics (Figure 3).

**Figure 4:**
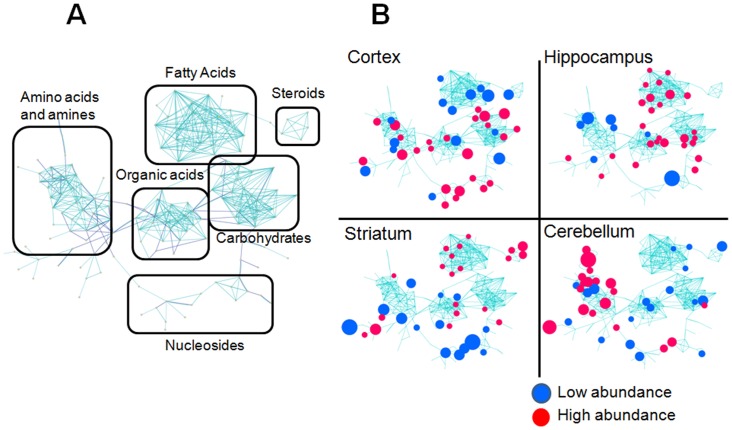
Distinct brain regions harbor discrete “signature” pools of non-glycolytic substrates for energy production. (**A**) Schematic representation of chemical classes grouping of metabolites from MetaMapp analysis. (**B**) Metabolic networks of biochemical reaction pairs (dark blue edges) and chemical similarity (light blue edges) show the regulation of all identified metabolites in four brain regions. Blue = down regulated metabolites, red = up regulated metabolites with a median false discovery rate <0.5% from SAM (n = 5 or 6). Ball sizes reflect magnitude of differential metabolite expression. Metabolites that were not significantly different were left unnamed in order to keep visual clarity.

We applied Hierarchical clustering analysis (HCA) using Spearman rank correlation [27] and average linkage methods [28] to evaluate the chemical classification of the region-specific metabolic pools (Figure 3B). Since they are functionally distinct, no one brain region can be taken as a “reference” point for the other regions. Thus, we developed a three-pronged quantile-based statistical strategy to identify significance of the region-specific substrate pools from a global perspective. In the first step, we pooled the metabolites from all four of the brain regions as a single group. Second, to generate a “global Gaussian distribution” we quantile-normalized the four groups of distribution to each other, without a reference distribution (Figure S4) using an open source software, geWorkbench [29]. In the third step, the region-specific signal intensities were calculated relative to the global distribution using Significance Analysis of Microarray (SAM) (Figure S5). The relative increases and decreases were assigned as red (high) or blue (low), respectively (Figure 4) (Figure S6, S7, S8, S9). Using this approach, each region was considered as a distinct component of the whole brain, and the unique features of the CTX, for example, could be deduced without the need for multiple pair-wise comparisons.

The region-specific metabolic pools were displayed using the published strategy based on MetaMapp [30], [31], a biochemical graphing approach that sorts according to biochemical reaction pair information and chemical similarity (Figure 4A). The reconstructed network consisted of six unique expression modules: fatty acids, steroids, carbohydrates, organics acids, amino acids, and nucleic acids (Figure 4B). The red and blue metabolite intensities in each brain region, which typically ranged from 2–6, were superimposed to generate a metabolic map, as illustrated for each brain region (Figure 4B) (enlarged with annotation in Figure S6, S7, S8, S9).

**Figure 5:**
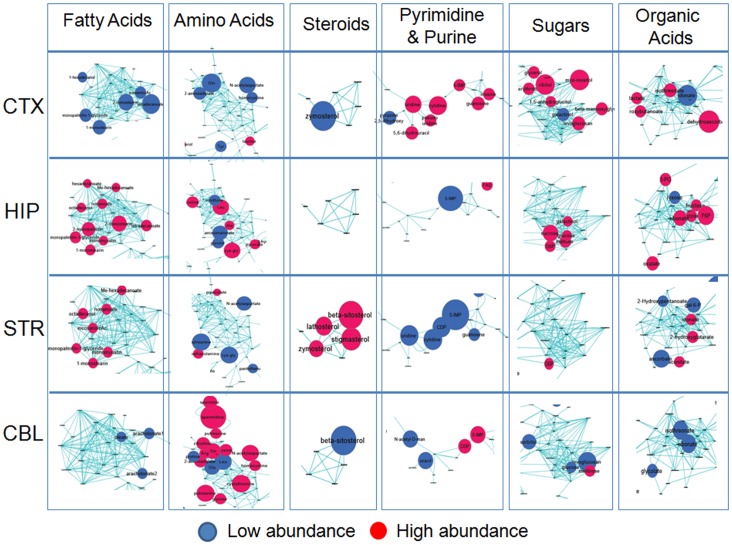
Magnified visual table of the observed six chemical classes in each region of the brain. The metabolic network is sub-categorized according to the resultant network topology. The clustered sub-network provides unique expression pattern of each brain region. Columns indicate six sub-clusters of the metabolic network and rows represent the four brain regions analyzed. Blue = down regulated metabolites, red = up regulated metabolites at a median false discovery rate <0.5% from SAM (n = 5 or 6). Ball sizes reflect magnitude of differential metabolite expression. Metabolites that were not significantly different were left unnamed in order to keep visual clarity.

Remarkably, each brain region had a distinct metabolic signature (Figure 4B). For ease of comparison, we individually magnified the six chemical groups from each region (Figure 4B) and enlarged with annotation (Figure S6, S7, S8, S9), and directly compared them in a visual table (Figure 5). For example, various fatty acids were statistically higher in the STR and the HIP, were lower in the CTX, and could not be distinguished as different relative to the global distribution in the CBL (Figure 5) (Figure S6, S7, S8, S9). The STR and HIP were, in many ways, similar, but the STR was enriched in the pool size of steroids and nucleic acids (Figure 5). A marked enrichment in amino acids and polyamines characterized the CBL, without obvious changes in other chemical groups (Figure 5). Although glucose is a requirement for brain metabolism, the products of glucose metabolism were strikingly region-specific, resulting in discrete pools of non-glycolytic substrates as resources for energy production (Figure 5, Figure S6, S7, S8, S9).

### Non-glycolytic Substrates Serve as a Region-specific Energy Source in Synaptosomes under Stress

Mitochondrial dysfunction confers a regional susceptibility to neuronal toxicity [1]–[4]. Thus, we tested whether use of the non-glycolytic pools conferred a region-specific response in mitochondrial metabolism in purified synaptosomes.

We artificially created an energy deficit by blocking oxidative phosphorylation with fluoro-carbonyl cyanide phenylhydrazone (FCCP) [32](Figure 2A and Figure S2). FCCP destroys the proton gradient in MT, which fails to convert electron flow into ATP, and OCR increases in response to the imposed stress (Figure 2A). The rise in OCR after FCCP treatment (referred to as mitochondrial spare respiratory capacity, SRC) reflects the maximum ability of MT to maintain energy production in response to the stress [33] (Figure 2A). OCR from STR, HIP, and CTX increased approximately 3–6 fold with SRC being the highest in the HIP (Figure 2B,C). The region-specific rise in OCR was reversible. Treatment with antimycin A (AA) [34] a blocker of complex III restored OCR to baseline, and all of the region-specific differences disappeared (Figure 2B). These findings indicated that the mitochondrial response to crisis was intrinsically different among synaptosomes. While synaptosomes were equally capable of substrate oxidation under basal conditions, the mitochondrial response to energy crisis was region-specific even under the condition of saturating glucose.

**Figure 6:**
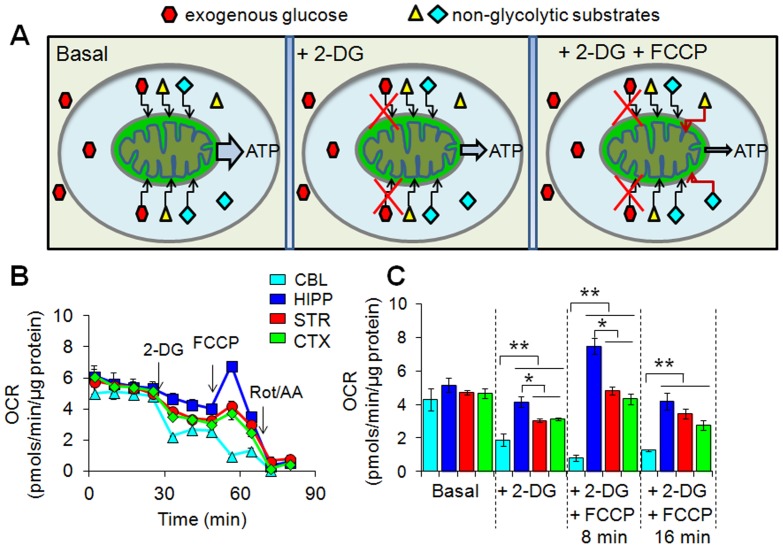
Energy deficits induce region-specific OCR that depends on use of non-glycolytic substrates in synaptosomes. (**A**) Schematic representation of experimental design for bioenergetics analysis of mitochondrial respiration on exogenous glucose and endogenous non-glycolytic substrates on various conditions. (**B**) Representative profiles of brain regional OCR under basal conditions and upon injections of 2-DG and FCCP. Each profile represents one independent biological experiment analyzed in triplicate. Data are means ± SEM (n = 3). The arrows indicate the injection of the inhibitors. Three independent experiments were performed to obtain quantification of OCR presented in (**C**). (**C**) Synaptosomal OCR under basal condition and upon inhibitions with 2-DG and FCCP. Upon inhibition with 2-DG, glycolysis is inhibited and energy arises only from endogenous pyruvate and non-glycolytic substrates. OCR is highest in the hippocampus, moderate in the striatum and cortex, and the lowest in the cerebellum. Upon FCCP injection after 2-DG, the same OCR pattern is observed. The OCR increase was short-lived and decreased at approximately 16 minutes upon FCCP treatment. Three independent experiments were performed. Data are means ± SEM (n = 3). *P<0.05, **P<0.01 with one-way ANOVA and Fisher’s LSD.

We tested whether non-glycolytic substrates supplied the energy source during stress. Synaptosomes were treated with 2-DG followed by addition of FCCP (Figure 6). Under these conditions, glucose is excluded as a substrate for oxidative phosphorylation, and OCR relies exclusively on endogenous non-glycolytic substrates (Figure 6A). When both glycolysis and oxidative phosphorylation were inhibited simultaneously for approximately 8 minutes (Figure 6B,C, 2-DG+FCCP, 8 minutes), OCR in the HIP, STR and CTX increased substantially relative to basal state or to 2-DG alone (Figure 6B,C, basal and +2-DG). Thus, the region-specific compensation for the FCCP-induced energy deficit did not depend on glycolysis under these conditions (Figure 6A). Moreover, the rise in OCR in synaptosomes treated with both 2-DG and FCCP displayed the same region-specific pattern as that of 2-DG (Figure 6B,C) or FCCP (Figure 2B,C) alone. These findings implied that the same pools of non-glycolytic substrates were used in both the basal and stressed states. Since the majority of ATP production was attenuated by the combined 2-DG and FCCP block, OCR decreased by 16 minutes after addition of FCCP, as the internal substrates were consumed (Figure 6B,C, 2-DG+FCCP, 16 minutes). Thus, use of non-glycolytic pools in synaptosomes conferred a region-specific response during crisis.

## Discussion

It has been extraordinarily difficult to establish how glucose is processed among brain regions, why glucose utilization in MT differs among brain regions in the resting state, and the basis for region-specific susceptibility in disease states. Our results provide fundamental insight into these long-standing issues. First, we demonstrate here that purified regional synaptosomes inherently differ in their metabolism (Figures 1 and 2). Non-glycolytic sources fuel the majority of OCR in the basal state of purified synaptosomes, even when glucose is saturating (Figure 2). This property predicts that glucose metabolism will differ among brain regions, as observed. Second, glucose metabolism results in discrete non-glycolytic pools that are “signatures” for the regions and are poised to fuel distinct responses. The mapping provides one of the first integrated views of glucose processing among brain regions (Figure 5). Third, OCR is similar in synaptosomes from all four brain regions in the resting brain, but heterogeneous contributions from non-glycolytic sources are unmasked during an energy crisis (Figures 2 and 6). Differences in basal metabolism provide a plausible basis for priming region-specific responses to an altered state.

Why distinct brain regions maintain different substrate pools is unknown. *In vivo,* astrocytes provide lactate to neurons as a major source of energy, and replenish the neuronal pool of glutamine and recycle glutamate [35]–[37]. However, we find that metabolism of purified synaptosomes is region-specific in the absence of astrocytes, implying that their differential respiration depends on internal non-glycolytic stores. MT simultaneously oxidize glutamate, pyruvate, as well as metabolites of the TCA cycle such as malate and succinate in activated neurons [38], but because glycolytic activity in neurons is low, other mechanisms to increase TCA intermediates are required. Thus, non-glycolytic stores are poised to play key roles in restructuring metabolism to meet the new demand. In cardiomyocytes, metabolic fluctuations stimulate gene expression of glycolytic enzymes that enhance the efficiency of glucose oxidation [39]. Specifically, expression of a new hexokinase isoform shifts the intracellular distribution of the enzyme from the cytosol to the MT. The translocation of hexokinase to the MT increases the kinetic efficiency of glycolysis and significantly increases ATP production and its delivery by phosphocreatine shuttling. All of this occurs despite a reduction in total glycolytic capacity in these cells. We have only incomplete information on the transcriptomes or the proteomes that operate in regions of the of mouse brain. However, such a model poises “signature” substrate pools as part of a highly adaptable glycolytic remodeling network to discharge ATP, and to supply substrates for maintenance of brain regions with different energetic competence [40], [41]. Differential substrate pools imply that metabolic restructuring is tailored to meet the needs of each region.

To our knowledge, the GC-MS approach described here includes the largest number of authentically identified metabolites, and yields one of the first comprehensive snapshots of region-specific brain metabolism on a global scale. The unique “signature” features of each brain region are obvious in the images (Figure 5). The chemical grouping technique provides an immediate picture of not only the metabolites but also the metabolic pathways that distinguish brain regions. For example, fatty acid metabolism characterizes the HIP (Figure 5). Furthermore, our statistical approach maximizes efficiency of the analysis by reducing the number of pair-wise comparisons among the four brain regions from six to one. The approach provides a useful baseline from which to evaluate any altered state (e.g., cancer, diabetes, or other metabolic disorders), and is applicable to any tissue.

The unique metabolic profiles generate testable hypotheses for how distinct brain regions might function. For example, the basal OCR of the CBL is similar to the other regions, yet the response of cerebellar synaptosomes to stress is strikingly different. Indeed, the GC-MS profiling reveals that the metabolic hallmark of the CBL is a high abundance of amino acids, a signature that is not shared with other brain regions (Figure 4, 5 and Figure S9). In the CBL, the need for glucose oxidation to meet basal respiration is higher (Figure 2D), but the ability of glycolysis to “fill-in” for oxidative phosphorylation (Figure 1E) is more limited relative to the other regions. From the viewpoint of bioenergetics, amines and amino acids provide carbon skeletal backbones suitable for both glucose and ketone bodies synthesis [42]. The high abundance of amino acids increases the glutamine pool size [43], spermine and spermidine are broken down to acetyl-CoA, and metabolism of N-acetylaspartate (NAA) leads to eventual metabolic conversion of NAA to α-ketoglutarate [44], all of which provide substrates that can be directly used in the TCA cycle. Thus, the CBL has multiple stable sources for ATP production, which by-pass glycolysis and do not depend on the slow breakdown of fats.

Many other factors will contribute to region-specific neuronal metabolism. Due to the large variation in glia-neuron ratios among regions, we focused only on region-specific neuronal metabolism. Thus, we cannot, as yet, precisely assign metabolites to cell types within a brain region. In our initial stages, we have limited the analysis to discrimination among well-defined brain structures. Finer dissection or separation techniques will be needed to distinguish, for example, the CA1 from the CA3 layers of the HIP. Nonetheless, we find that basal metabolism and unique pools of non-glycolytic substrates provide fundamentally different abilities of regional synaptosomes to mount a response to energy crisis. In disease states, we envision a model in which region-specific susceptibility stems from metabolism of non-glycolytic substrates, that limits the extent to which neurons respond to the stress. Glucose levels vary daily before and after meals, are low during a starvation [45], and are often diminished in disease states [46]–[48]. Thus, substrate diversity is likely to provide an inherent advantage in maintaining energetic consistency under variable conditions.

## Methods

### Animals

We used C57BL/6 mice. All procedures involving animals were performed in accordance with the National Institutes of Health Guide for the Care and Use of Laboratory Animals. Protocols were approved by the Lawrence Berkeley National Laboratories Animal Welfare and Research Committee.

### Preparation of Brain Regional Synaptosomes

Brain regional synaptosomes were isolated as described previously [20], [21] with slight modifications. Briefly, after rapid decapitation using a guillotine, the mouse brain was quickly extracted, rinsed with fresh ice-cold homogenization buffer (320 mM sucrose, 1 mM EDTA, 5 mM Tris, 0.25 mM dithiothreitol, pH 7.4). The cerebellum, hippocampus, striatum and cortex were obtained after immediate dissection on a homogenization buffer-filled petri dish laid on an ice bath. Dissected brain regions were quickly transferred to a 15 mL pre-chilled Dounce glass containing 1 mL (for striatum, hippocampus, and cerebellum) or 3 mL (for cortex) of homogenization buffer and gently homogenized with 8–10 up-and-down strokes. The homogenate was centrifuged at 1,000×g for 10 minutes at 4°C. The supernatant was carefully layered on a freshly prepared discontinuous Percoll gradient of 3%, 10%, and 23% (from top to bottom) and centrifuged at 32,500×g for 8 minutes at 4°C (JA-17 fixed angle rotor, Beckman). Synaptosomes were obtained as the band between the 10% and 23% Percoll interface. The synaptosomal fraction was then diluted in the homogenization buffer and centrifuged at 15,000×g for 15 minutes at 4°C to remove the Percoll solution and obtain the final synaptosomal pellet. Protein concentration of the synaptosomal pellet was determined using the Bio-Rad Bradford assay (BioRad Laboratories).

### Quality Control of Brain Regional Synaptosomes

To ensure quality and consistency of the brain regional synaptosomes preparations, we instituted the following protocols. All the four brain regions were processed from the same animal, and the synaptosomes from each region were isolated in parallel on the same day. Bioenergetic parameters from synaptosomes of all four regions were measured together on the same instrument at the same time using the same reagents in each run. Thus, any prep-to prep variation was normalized. All measures were made in at least three independent preparations. Data are never used unless they were reproducible in three experiments. The synaptosomal protein concentrations were determined each time to track the consistence of preparation for each region. The same amount of synaptosomes (6 µg/well) was plated in each experiment, and we checked protein levels to ensure equal numbers from each region. To further evaluate the viability and consistency, we checked the isolated synaptosomes with Mitotracker DeepRed staining to establish that the preparations were viable and the MT were active. Using a plate reader, we observe no detectable difference in MitoTracker DeepRed signal intensity in synaptosomes among the four brain regions.

### Measurement of Extracellular Acidification Rate (ECAR) and Oxygen Consumption Rate (OCR)

ECAR and OCR were obtained by using a Seahorse XF96 Extracellular Flux Analyzer (Seahorse Bioscience, Billerica, MA) [49]. Freshly isolated synaptosomal pellets were immediately and gently re-suspended in the synaptosomal assay solution (SAS, 3.5 mM KCl, 120 mM NaCl, 1.3 mM CaCl_2_, 0.4 mM KH_2_PO4, 1.2 mM Na_2_SO4, 2 mM MgSO4, 15 mM D-glucose, and 4 mg/ml BSA, pH 7.2 at 37°C) on ice. 150 µL suspensions of synaptosomes (6 µg protein/well) were plated on a pre-chilled Seahorse PS 96-well microplate (Seahorse Bioscience, Billerica, MA) [49]. The plate was centrifuged at 3,220×g for 50 minutes at 4°C, subsequently incubated in 37°C (without CO_2_) for 15 minutes, and then transferred to the XF flux analyzer for respiration measurement. The measurement cycle consisted of a 3 minutes mixing time and a 4 minutes measurement time. After four basal measurements, 2 µg/mL oligomycin (inhibitor of ATP synthase) or 100 mM 2-deoxy-glucose (2-DG) was injected and three measurement cycles were performed. Subsequently, 4 µM carbonylcyanide p-trifluoromethoxyphenylhydrazone (FCCP) (an optimized concentration to give maximum respiratory capacity) was injected followed by an addition of a cocktail of 2 µM rotenone and 2 µM Antimycin A. Two measurement cycles were performed for each compound injection. Each experimental point is an average of a minimum of three replicate wells and each experiment was performed with a minimum of three biological replicates. In each individual experiment, two mice per group were utilized.

### GC-TOF MS Analysis for Metabolites

Following decapitation, the brain regions (cortex, striatum, hippocampus, cerebellum) were isolated, snap-frozen with liquid nitrogen, and freeze-dried until analysis [50], [51]. Briefly, lyophilized cells were disrupted using a single 5 mm i.d. steel ball, followed by the addition of 0.75 mL extraction solvent of methanol:isopropanol:water (3∶3:2) and vortexing. After 5 min centrifugation at 16,100 g, 0.70 mL extracts were collected and concentrated to dryness for further analysis. A mixture of internal retention index (RI) markers was made using fatty acid methyl esters of C8, C9, C10, C12, C14, C16, C18, C20, C22, C24, C26, C28 and C30 in chloroform at a concentration of 0.8 mg/ml (C8–C16) and 0.4 mg/ml (C18–C30). One microliter of this RI mixture was added to ten microliters of a solution containing 40 mg/ml of 98% methoxyamine hydrochloride (CAS No. 593-56-6, Sigma, St. Louis, MO) in pyridine (silylation grade, Pierce, Rockford, IL), and incubated at 30°C, 800 rpm for 90 min (ThermoStat plus, Eppendorf North America Inc, San Diego CA). Ninety microliters of MSTFA containing 1%TMCS (1 ml bottles, Pierce, Rockford, IL) was added and shaken at 37°C for 30 min. The derivatized sample was managed using a Gerstel automatic liner exchange system with multipurpose sample MPS2 dual rail controlled by Maestro software for injection of the 0.5 µl sample to a Gerstel CIS cold injection system (Gerstel, Muehlheim, Germany). The injector was operated in splitless mode; the split vent was opened after 25 s. Samples were injected into the 50°C injector port, which was ramped to 250°C in 12°C/min and held for 3 min. Volatilized metabolites were separated using an Agilent 6890 gas chromatograph (Santa Clara, CA), managed by Leco ChromaTOF software (St. Joseph, MI). The GC was equipped with a 30 m long, 0.25 mm i.d. Rtx5Sil-MS column (Restek, Bellefonte, PA, USA), 0.25 mm 5% diphenyl film and additional 10 m integrated guard column). The temperature was held at 50°C for 1 min and then ramped at 20°C min^−1^ to 330°C where it was retained for 5 min. Mass spectrometry was performed by a Leco Pegasus III time of flight mass spectrometer (St. Joseph, MI) with 250°C transfer line temperature, electron ionization at −70 eV and an ion source temperature of 280°C. Mass spectra were acquisitioned from *m/z* 85 to 500 at 17 spectra s^−1^ and 1850 V detector voltage. Result files were preprocessed directly after data acquisition and stored as ChromaTOF-specific *.peg files, as generic *.txt result files and additionally as generic ANDI MS *.cdf files [31], [50]. Metabolite identifications were made based on spectral similarity and retention time index using BinBase and were matched against the Fiehn mass spectral library of ∼1,200 authentic metabolite spectra using retention index and mass spectrum information or the NIST05 commercial library (http://fiehnlab.ucdavis.edu/Metabolite-Library-2007/) [51]. Identified metabolites were reported if present within at least 50% of the samples per study design group [52].

### Statistical Data Analysis

Brain tissues were isolated and extracted (cerebellum: hippocampus : striatum : cortex, 0.5∶1:1∶0.1 volume). We normalized the data set by using quantile normalization method (see text) [53], [54], which is frequently used in microarray data analysis, to transform the identical distributions of datasets (Figure S4). Following the normalization procedure, we validated the region-specific metabolites using two independent approaches referred to as statistic one and statistic two. In the first approach, we evaluated the individual metabolites that deviated from the global Gaussian distribution using two group-Significance Analysis of Microarray (SAM) (statistics one, Figure S5 and Table S2 in File S1). Each brain region was independently evaluated for statistically different metabolites to form a profile for the region. This analysis provided comparison of the unique metabolic pool in each region. In statistics two, metabolites in any one region were aligned based on intensity, and the top 35 most significantly different metabolites were selected based on SAM [55], built in MultiExperimental Viewer (MeV 4.8.1 version) [56], generating a pool of metabolites that were significantly higher or significantly lower than the average (Table S3 in File S1). The analysis creates a list of metabolites with a threshold in which (0%<median false discovery rate <0.5%). The analysis resulted in the overview of the basal expression pattern of endogenous metabolites, indicated by the “out bounds” metabolites that characterized a particular region (Figure S5). The analyses were crosschecked for overlap to provide insight into the degree of false positives. Interestingly, the membership detected by the analysis in statistics one and statistics two was very similar, indicating that the metabolic features were robust (84% identity). In a one-way ANOVA, group A might be distinct from Group B but similar to group D, and expressing this relationship graphically is difficult. Thus, for visualization of significant metabolites, we did not need to express the fold change and p-value based on one specific brain region. The strategy provided statistical significance without the need for relative comparisons. The method also facilitated the graphical efficiency in the network structure.

For general statistics, the analyses were performed on all continuous variables using the Statistica software vs. 8.0 (StatSoft, Tulsa OK). Univariate statistics for multiple study design classes was performed by t-tests and one-way analysis of variance. Data distributions were displayed by box-whisker plots, giving the arithmetic mean value for each category, the standard error as box and whiskers for 1.96 times the category standard error. Clustering analysis was performed by hierarchical clustering analysis within Multi Experimental Viewer (TIGR) and multivariate statistics was performed by supervised partial least square (PLS) statistics within the Statistica data miner 8.0 vs software. The metabolic features of each brain region were separated by the combination of two linear discriminant vectors by Partial Least Squares (PLS) analysis. Vectors in the supervised linear dimension were ordered by the degree of variance in metabolite abundance. Vector 1 (30.5% total explained variance) discriminated metabolite profiles of STR and HIP from CTX and CBL (Figure 3A), while vector 2 (6.0% total explained variance) primarily separated clusters between STR and HIP.

### Metabolic Network

The metabolic network was constructed following methods developed by Fiehn and coworkers [30]. Briefly, Molefile [57] encoded chemical structures of all the identified metabolites were retrieved from PubChem Compound database [58] using compound identifiers (CIDs) and the NCBI Batch Entrez utility [59]. The retrieved structures were clustered using an online structural clustering tool at PubChem website. The tool uses Tanimoto chemical similarity co-efficient [60](range 0.0 to 1.0, where high score means high similarity between two metabolites) for similarity computation. The similarity matrix and the list of associated metabolites that were found in the KEGG biochemical database were incorporated as an input into the MetaMapp software for generation of Cytoscape [61] network files in simple interaction format (.SIF). A threshold of 0.7 Tanimoto score was used to define the similarity cut-off among metabolites. A KEGG Rpair [62] reaction network graph was created using a single-metabolic step neighbor finding algorithm in MetaMapp. The final network graphs were imported into Cytoscape and merged into a single network graph. Results of differential statistics were converted into Cytoscape node attribute files, and were imported into Cytoscape. The graph was visualized using an organic layout algorithm in Cytoscape. Fold change was mapped to node size, and direction (up/down) was visualized to node color (red/blue resp.). Metabolites not passing the statistical criteria remained invisible size. Metabolic mapping and its visualization are provided in supplement section as Cytoscape session file.

## Supporting Information

Figure S1 **GC/MS profiles of glycolytic intermediates.** The abundance of glycolytic intermediates among four different brain regions is measured using gas-chromatography mass spectrometry (GC/MS) (n = 5 or 6) (cerebellum: till, hippocampus: blue, striatum: red, cortex: light green). Data are displayed by box-whisker plots, giving the arithmetic mean for each category, the standard error as a box, and whiskers for 1.96 times the category standard error to indicate the 95% confidence intervals.
(TIF)

Figure S2 **Simplified diagram of glycolysis and oxidative phosphorylation and inhibitors.** Glycolysis converts glucose to cytosolic pyruvate, which is either converted to lactic acid or enters the mitochondrial matrix as a substrate for oxidative phosphorylation, measured as oxygen consumption rate (OCR, red). Rotenone, antimycin A, and oligomycin are the inhibitors of Complex I, Complex III, and ATP synthase, respectively. Fluoro-carbonyl cyanide phenylhydrazone (FCCP) is an ionophore, which allows re-entry of the protons into the mitochondrial matrix and dissipates the proton gradient. OCR is determined by using the Seahorse XF Flux Analyzer.
(TIF)

Figure S3 **GC/MS profiles of TCA cycle intermediates.** The abundance of TCA cycle intermediates among four different brain regions is measured using gas-chromatography mass spectrometry (GC/MS) (n = 5 or 6) (cerebellum: till, hippocampus: blue, striatum: red, cortex: light green). Data are displayed by box-whisker plots, giving the arithmetic mean for each category, the standard error as a box, and whiskers for 1.96 times the category standard error to indicate the 95% confidence intervals.
(TIF)

Figure S4 **Mass spectrometry data transformation. (A)** The distribution of metabolite intensities after logarithmic normalization. **(B)** The distribution of metabolite intensities following quantile normalization. X and Y-axis indicate frequency and normalized abundances of metabolites respectively.
(TIF)

Figure S5 **Identification of metabolites with significant differences in abundance using Significance Analysis of Microarray.** Scatter plot of the observed relative difference versus the expected relative difference. The solid line indicates the line for the condition, where the observed relative difference is identical to the expected relative difference. The two dotted lines display the region within +/− delta units from the “observed = expected” line. Delta is a vertical distance from the solid line of slope 1. The metabolites whose plot values are represented by black dots are regarded non-significant, those colored red have positive significance, and the green ones have negative significance.
(TIF)

Figure S6 **Relative pool size of primary metabolites of the cortex region.** The metabolic network shows relative abundance of primary metabolites of the cortex compared to those of other brain regions. Blue = down regulated metabolites, red = up regulated metabolites at a median false discovery rate <0.5% from SAM (n = 5 or 6). Ball sizes reflect magnitude of differential metabolite expression. Metabolites that were not significantly different were left unnamed in order to keep visual clarity.
(TIF)

Figure S7 **Relative pool size of primary metabolites of the hippocampus region.** The metabolic network shows relative abundance of primary metabolites of the cortex compared to those of other brain regions. Blue = down regulated metabolites, red = up regulated metabolites at 0%<median false discovery rate <0.5% from SAM (n = 5 or 6). Ball sizes reflect magnitude of differential metabolite expression. Metabolites that were not significantly different were left unnamed in order to keep visual clarity.
(TIF)

Figure S8 **Relative pool size of primary metabolites of the striatum region.** The metabolic network shows relative abundance of primary metabolites of the cortex compared to those of other brain regions. Blue = down regulated metabolites, red = up regulated metabolites at 0%<median false discovery rate <0.5% from SAM (n = 5 or 6). Ball sizes reflect magnitude of differential metabolite expression. Metabolites that were not significantly different were left unnamed in order to keep visual clarity.
(TIF)

Figure S9 **Relative pool size of primary metabolites of the cerebellum region.** The metabolic network shows relative abundance of primary metabolites of the cortex compared to those of other brain regions. Blue = down regulated metabolites, red = up regulated metabolites at 0%<median false discovery rate <0.5% from SAM (n = 5 or 6). Ball sizes reflect magnitude of differential metabolite expression. Metabolites that were not significantly different were left unnamed in order to keep visual clarity.
(PDF)

File S1 **Contains: Table S1, S2, S3.**
(XLSX)
