# Supplementary figures and images for: Distinct Pools of Non-Glycolytic Substrates Differentiate Brain Regions and Prime Region-Specific Responses of Mitochondria
Source: PLoS One. 2013 Jul 17;8(7):e68831. doi: 10.1371/journal.pone.0068831 (PMC3714274; doi:10.1371/journal.pone.0068831)

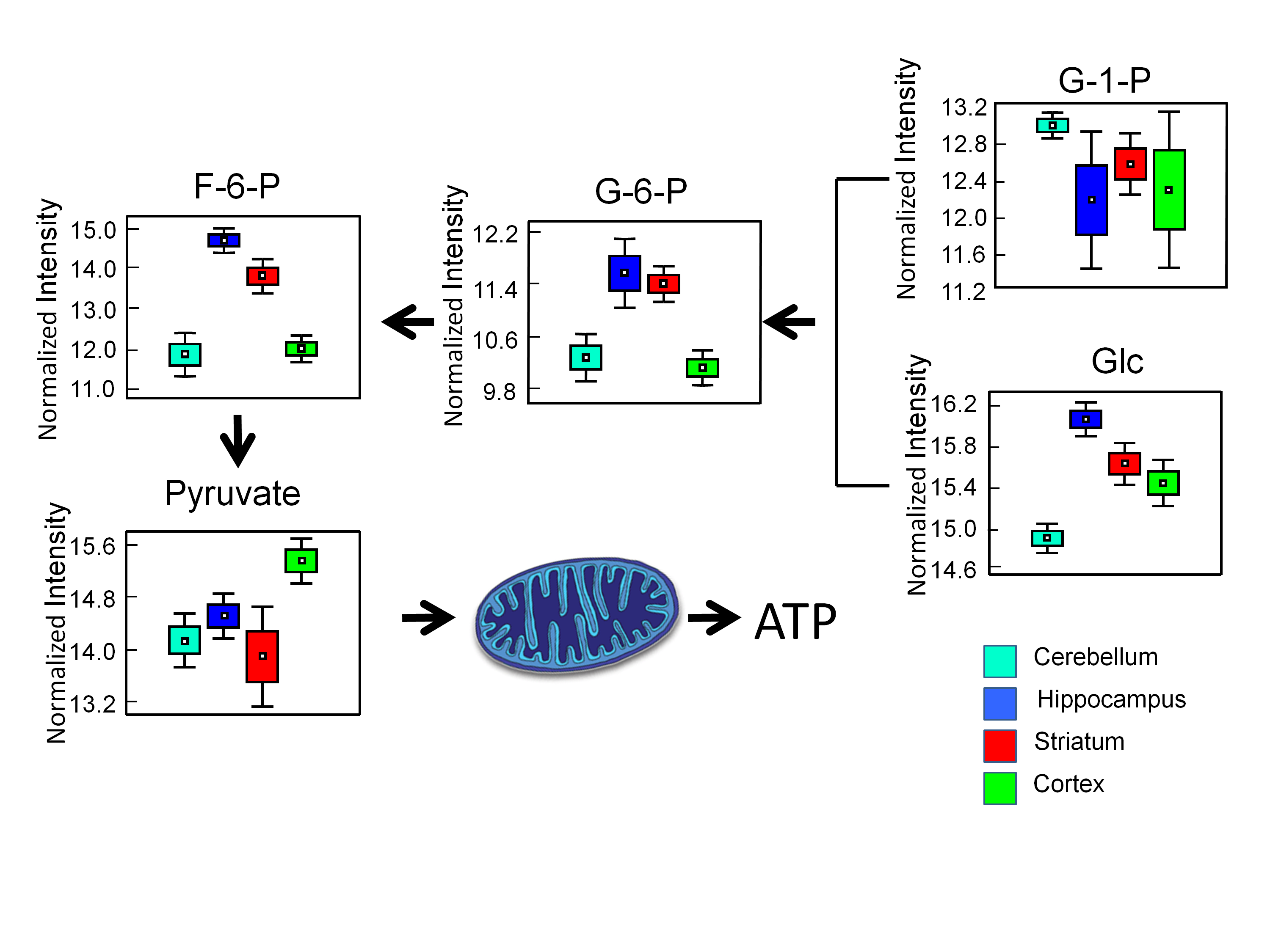

Supplement: Figure S1 — GC/MS profiles of glycolytic intermediates. The abundance of glycolytic intermediates among four different brain regions is measured using gas-chromatography mass spectrometry (GC/MS) (n = 5 or 6) (cerebellum: till, hippocampus: blue, striatum: red, cortex: light green). Data are displayed by box-whisker plots, giving the arithmetic mean for each category, the standard error as a box, and whiskers for 1.96 times the category standard error to indicate the 95% confidence intervals. (TIF) [file pone.0068831.s001.tif]

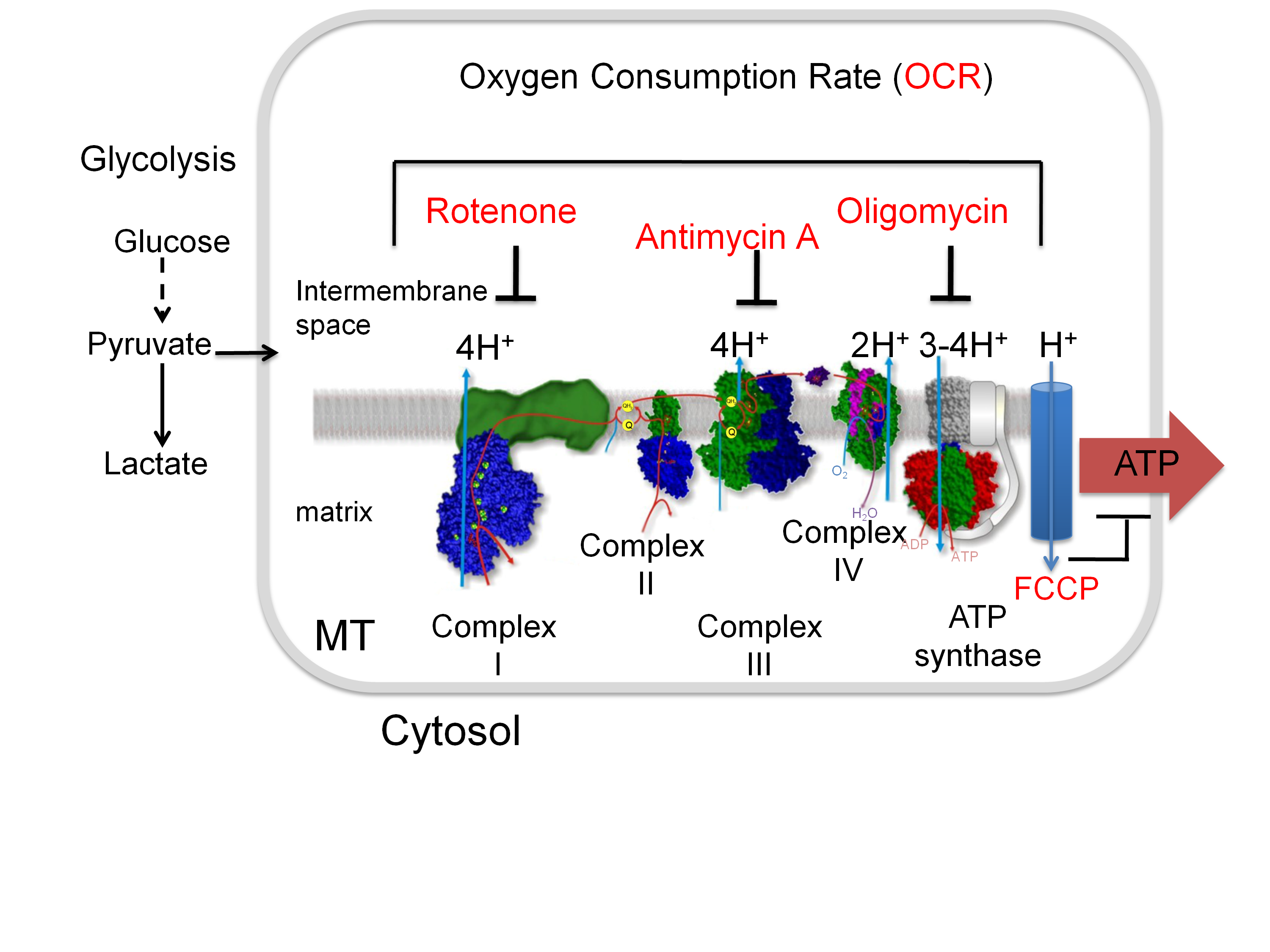

Supplement: Figure S2 — Simplified diagram of glycolysis and oxidative phosphorylation and inhibitors. Glycolysis converts glucose to cytosolic pyruvate, which is either converted to lactic acid or enters the mitochondrial matrix as a substrate for oxidative phosphorylation, measured as oxygen consumption rate (OCR, red). Rotenone, antimycin A, and oligomycin are the inhibitors of Complex I, Complex III, and ATP synthase, respectively. Fluoro-carbonyl cyanide phenylhydrazone (FCCP) is an ionophore, which allows re-entry of the protons into the mitochondrial matrix and dissipates the proton gradient. OCR is determined by using the Seahorse XF Flux Analyzer. (TIF) [file pone.0068831.s002.tif]

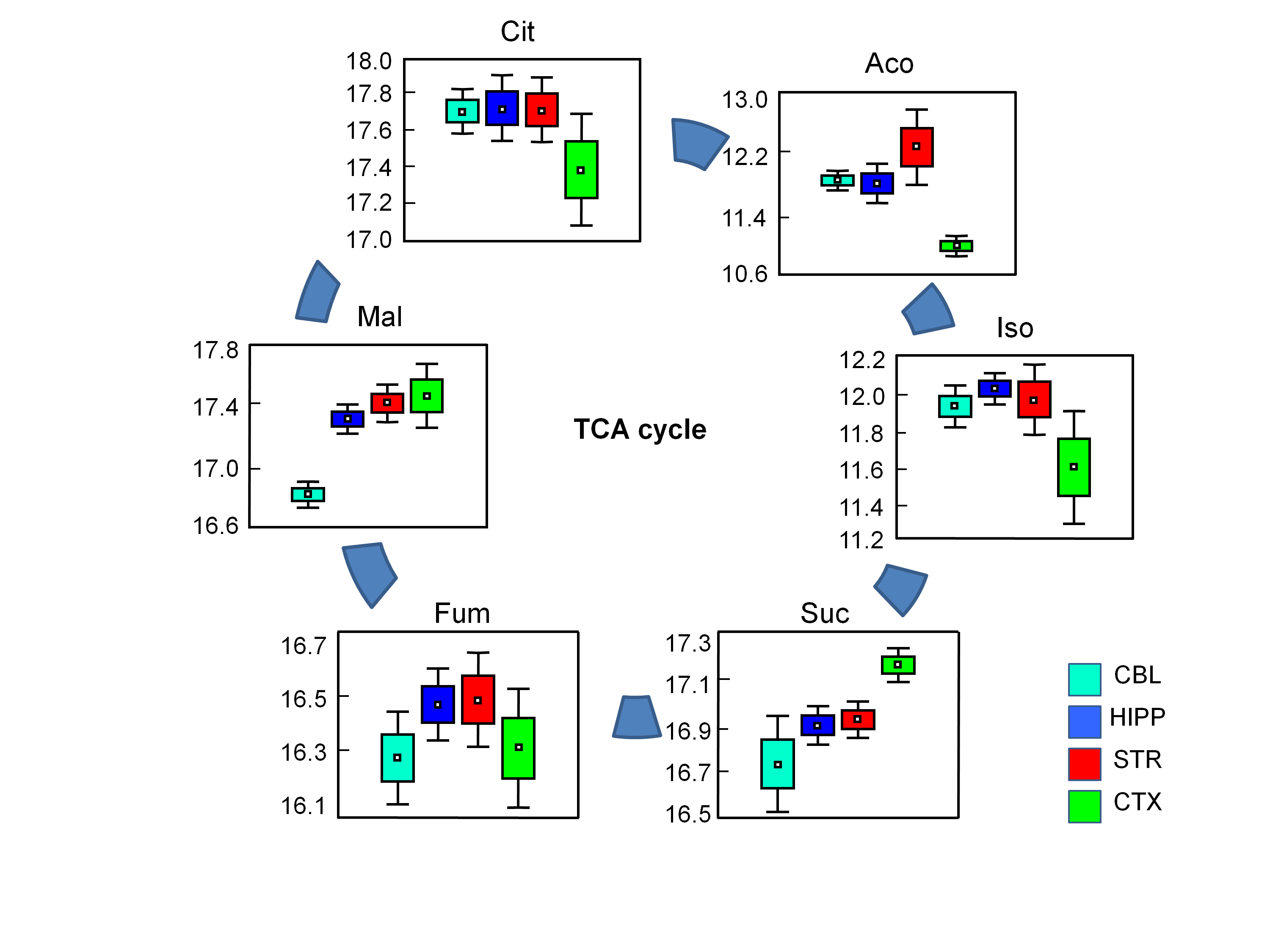

Supplement: Figure S3 — GC/MS profiles of TCA cycle intermediates. The abundance of TCA cycle intermediates among four different brain regions is measured using gas-chromatography mass spectrometry (GC/MS) (n = 5 or 6) (cerebellum: till, hippocampus: blue, striatum: red, cortex: light green). Data are displayed by box-whisker plots, giving the arithmetic mean for each category, the standard error as a box, and whiskers for 1.96 times the category standard error to indicate the 95% confidence intervals. (TIF) [file pone.0068831.s003.tif]

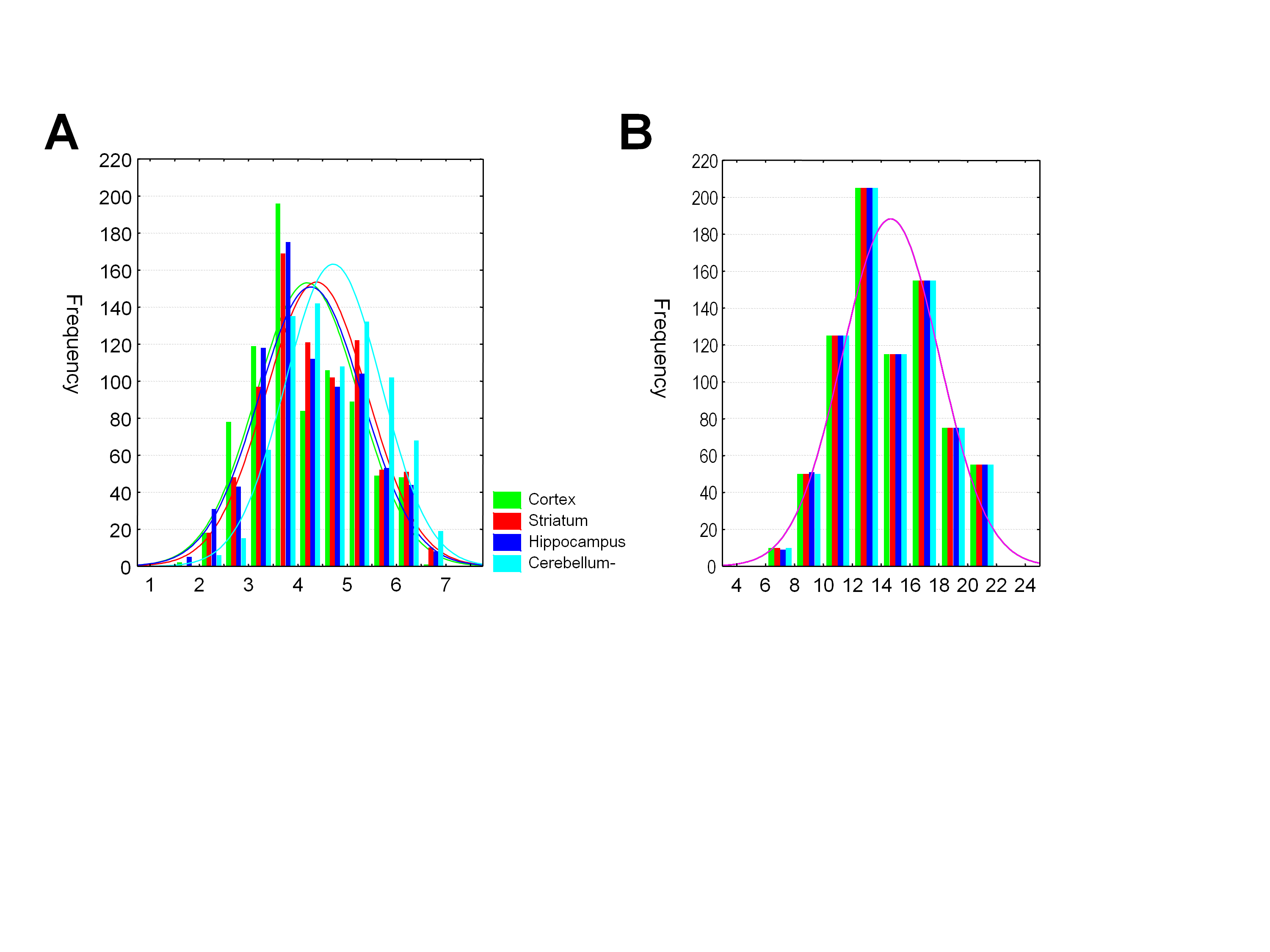

Supplement: Figure S4 — Mass spectrometry data transformation. (A) The distribution of metabolite intensities after logarithmic normalization. (B) The distribution of metabolite intensities following quantile normalization. X and Y-axis indicate frequency and normalized abundances of metabolites respectively. (TIF) [file pone.0068831.s004.tif]

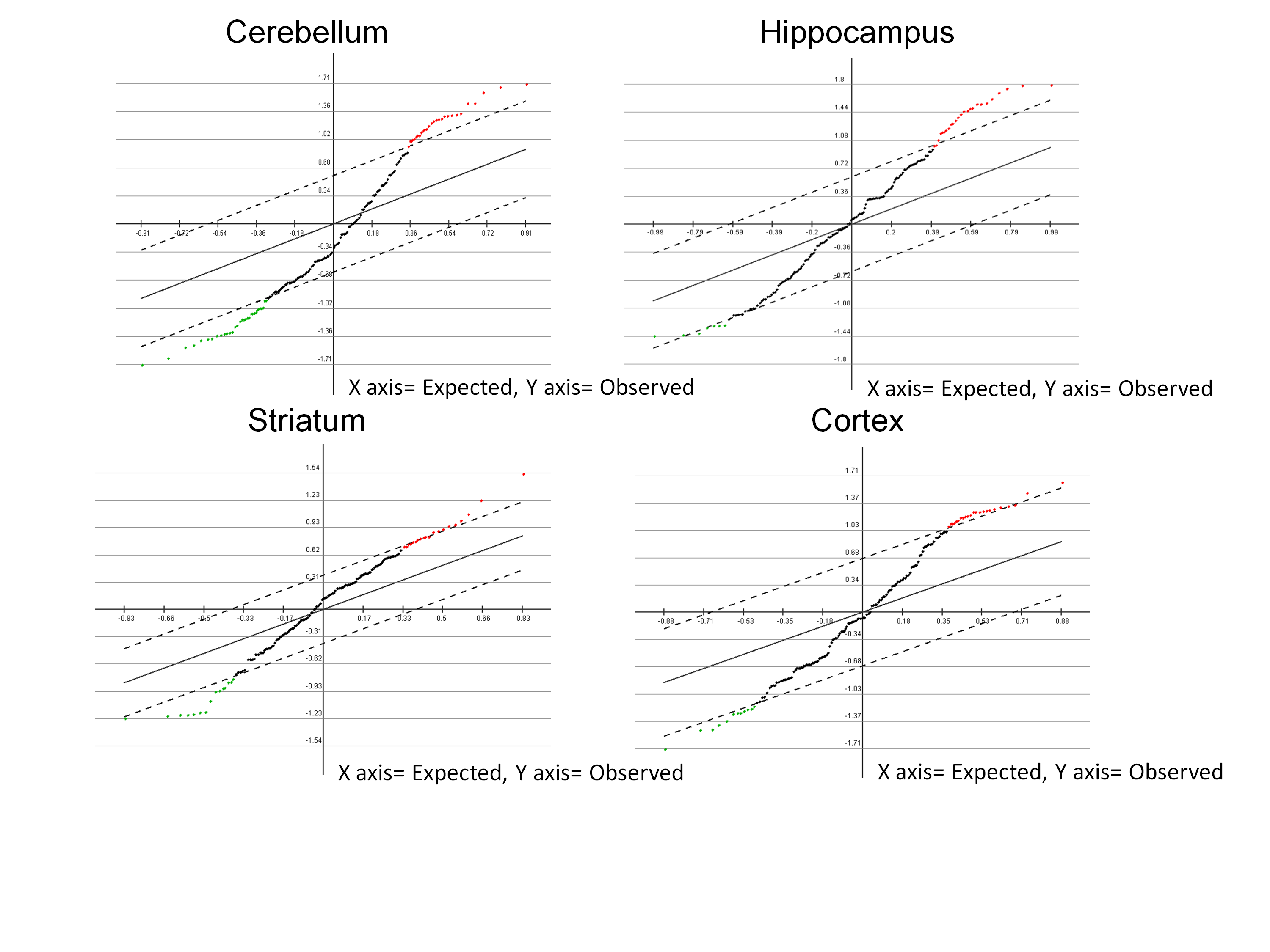

Supplement: Figure S5 — Identification of metabolites with significant differences in abundance using Significance Analysis of Microarray. Scatter plot of the observed relative difference versus the expected relative difference. The solid line indicates the line for the condition, where the observed relative difference is identical to the expected relative difference. The two dotted lines display the region within +/− delta units from the “observed = expected” line. Delta is a vertical distance from the solid line of slope 1. The metabolites whose plot values are represented by black dots are regarded non-significant, those colored red have positive significance, and the green ones have negative significance. (TIF) [file pone.0068831.s005.tif]

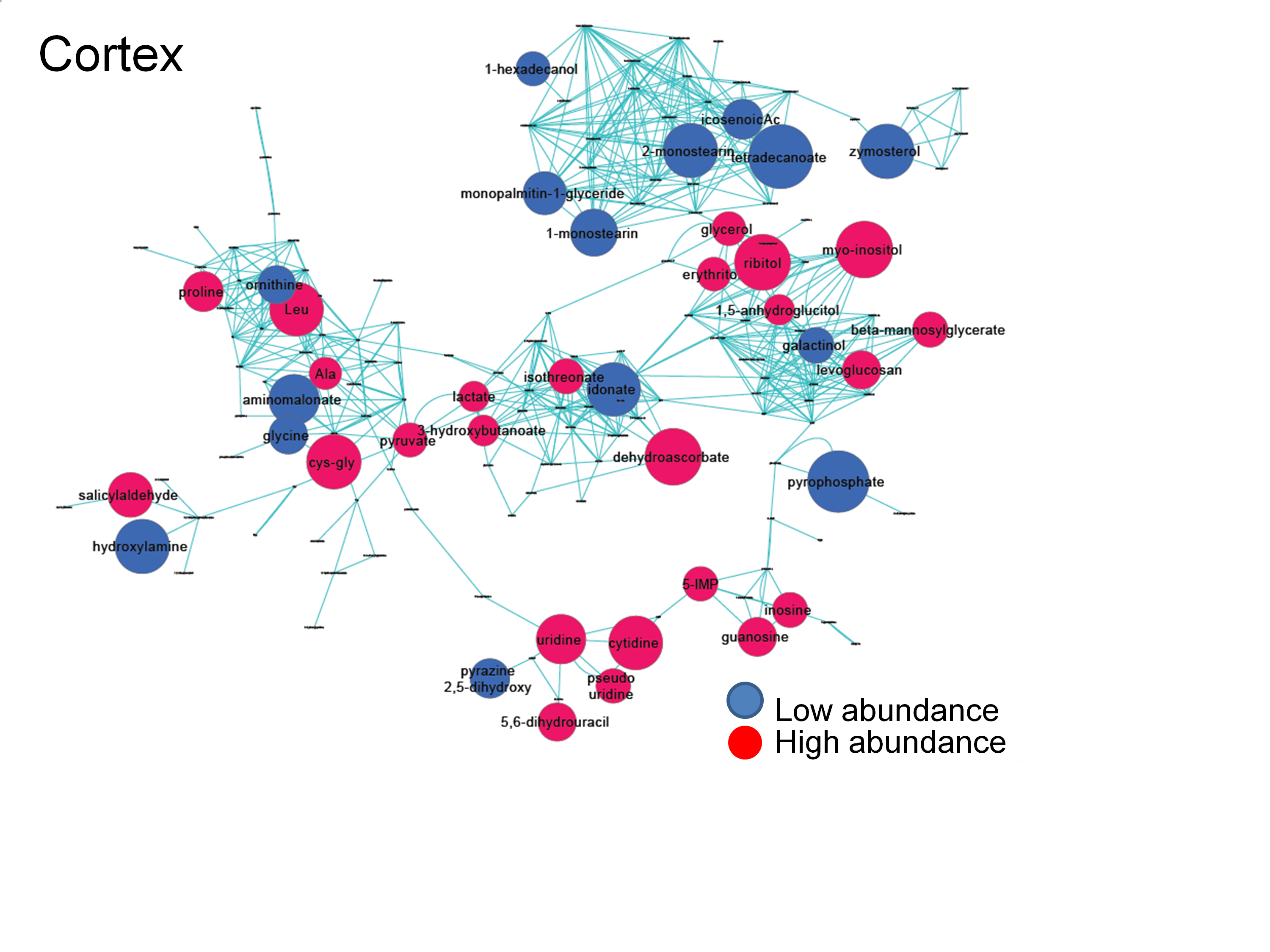

Supplement: Figure S6 — Relative pool size of primary metabolites of the cortex region. The metabolic network shows relative abundance of primary metabolites of the cortex compared to those of other brain regions. Blue = down regulated metabolites, red = up regulated metabolites at a median false discovery rate <0.5% from SAM (n = 5 or 6). Ball sizes reflect magnitude of differential metabolite expression. Metabolites that were not significantly different were left unnamed in order to keep visual clarity. (TIF) [file pone.0068831.s006.tif]

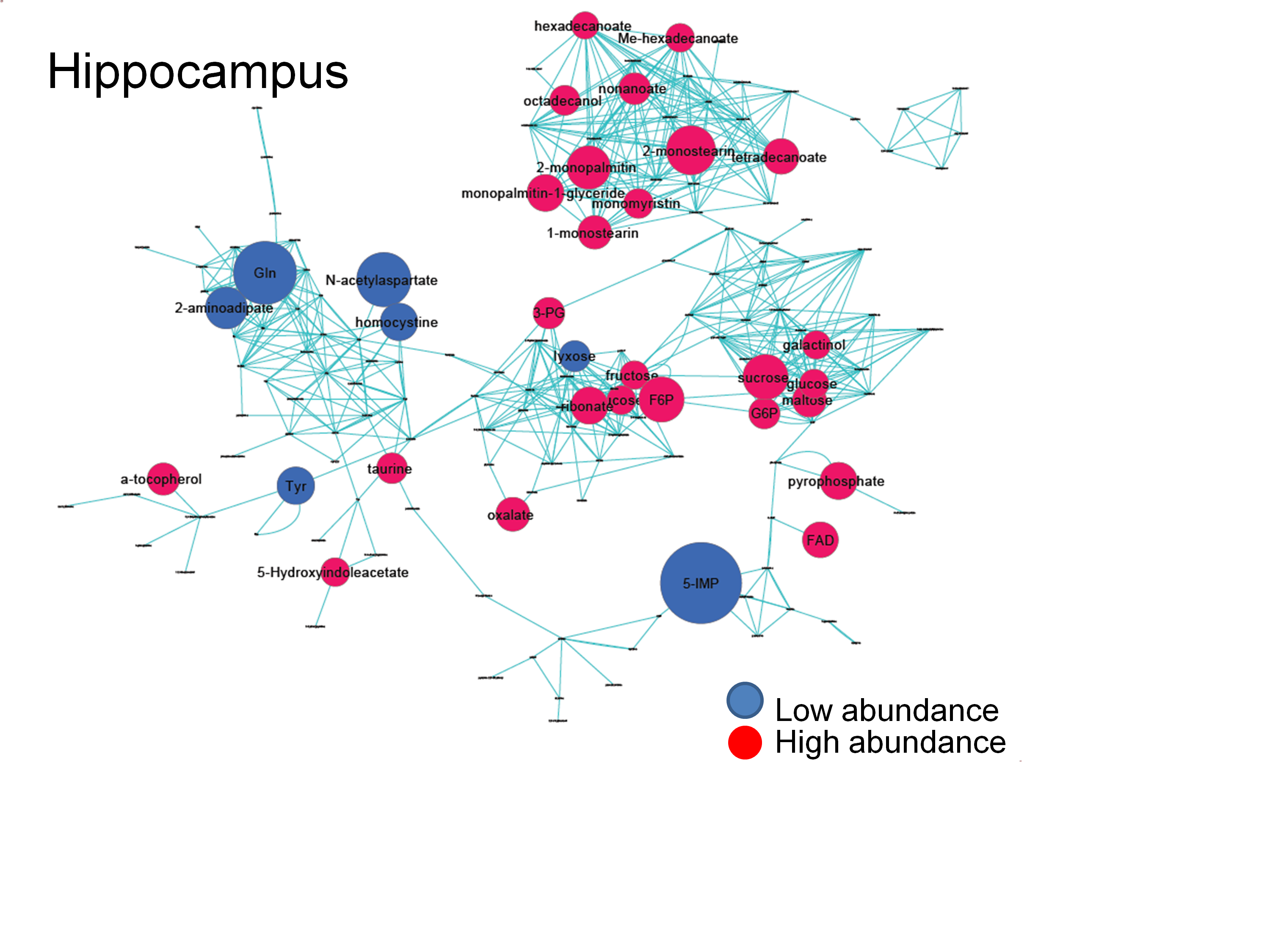

Supplement: Figure S7 — Relative pool size of primary metabolites of the hippocampus region. The metabolic network shows relative abundance of primary metabolites of the cortex compared to those of other brain regions. Blue = down regulated metabolites, red = up regulated metabolites at 0%<median false discovery rate <0.5% from SAM (n = 5 or 6). Ball sizes reflect magnitude of differential metabolite expression. Metabolites that were not significantly different were left unnamed in order to keep visual clarity. (TIF) [file pone.0068831.s007.tif]

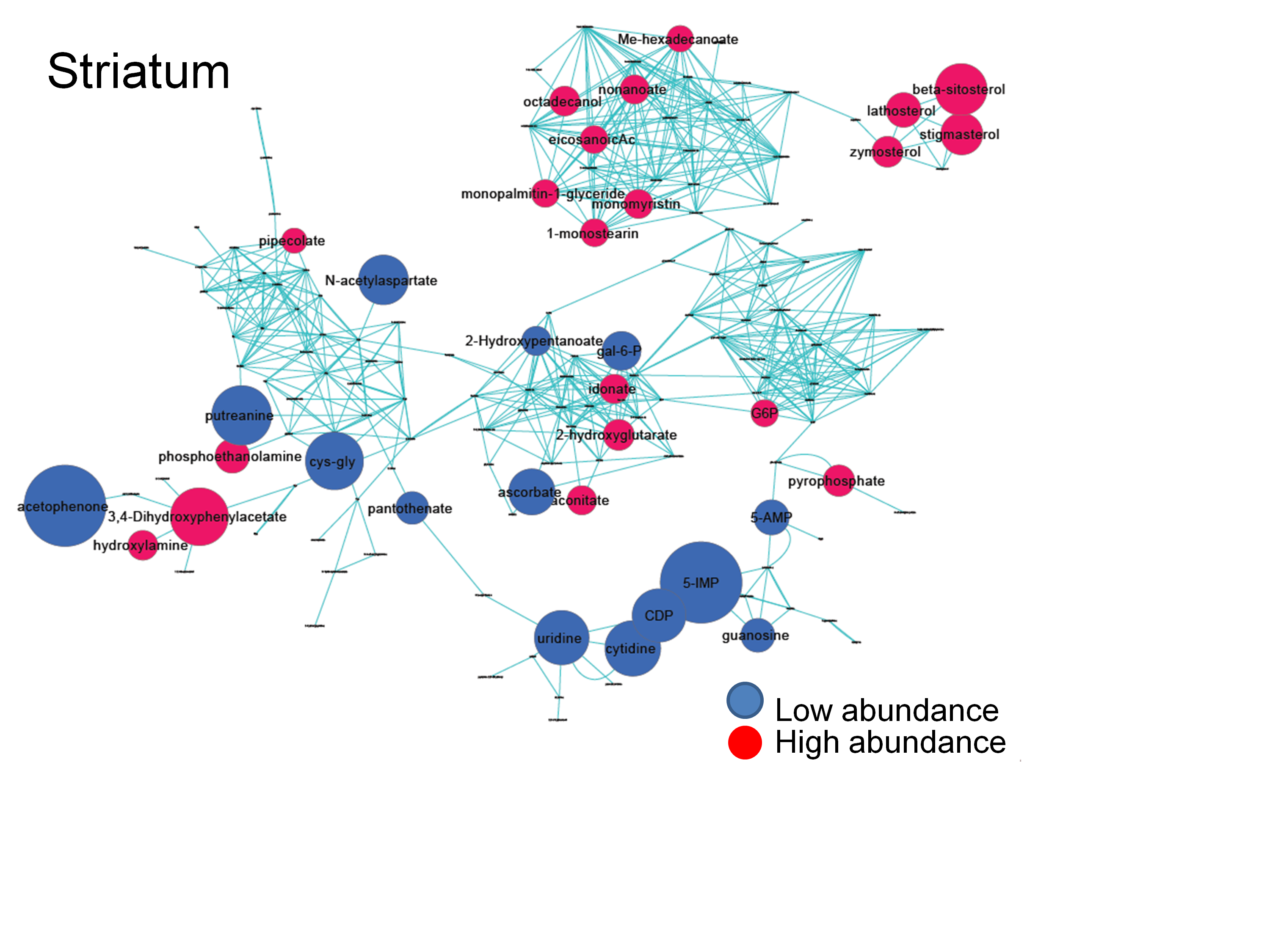

Supplement: Figure S8 — Relative pool size of primary metabolites of the striatum region. The metabolic network shows relative abundance of primary metabolites of the cortex compared to those of other brain regions. Blue = down regulated metabolites, red = up regulated metabolites at 0%<median false discovery rate <0.5% from SAM (n = 5 or 6). Ball sizes reflect magnitude of differential metabolite expression. Metabolites that were not significantly different were left unnamed in order to keep visual clarity. (TIF) [file pone.0068831.s008.tif]

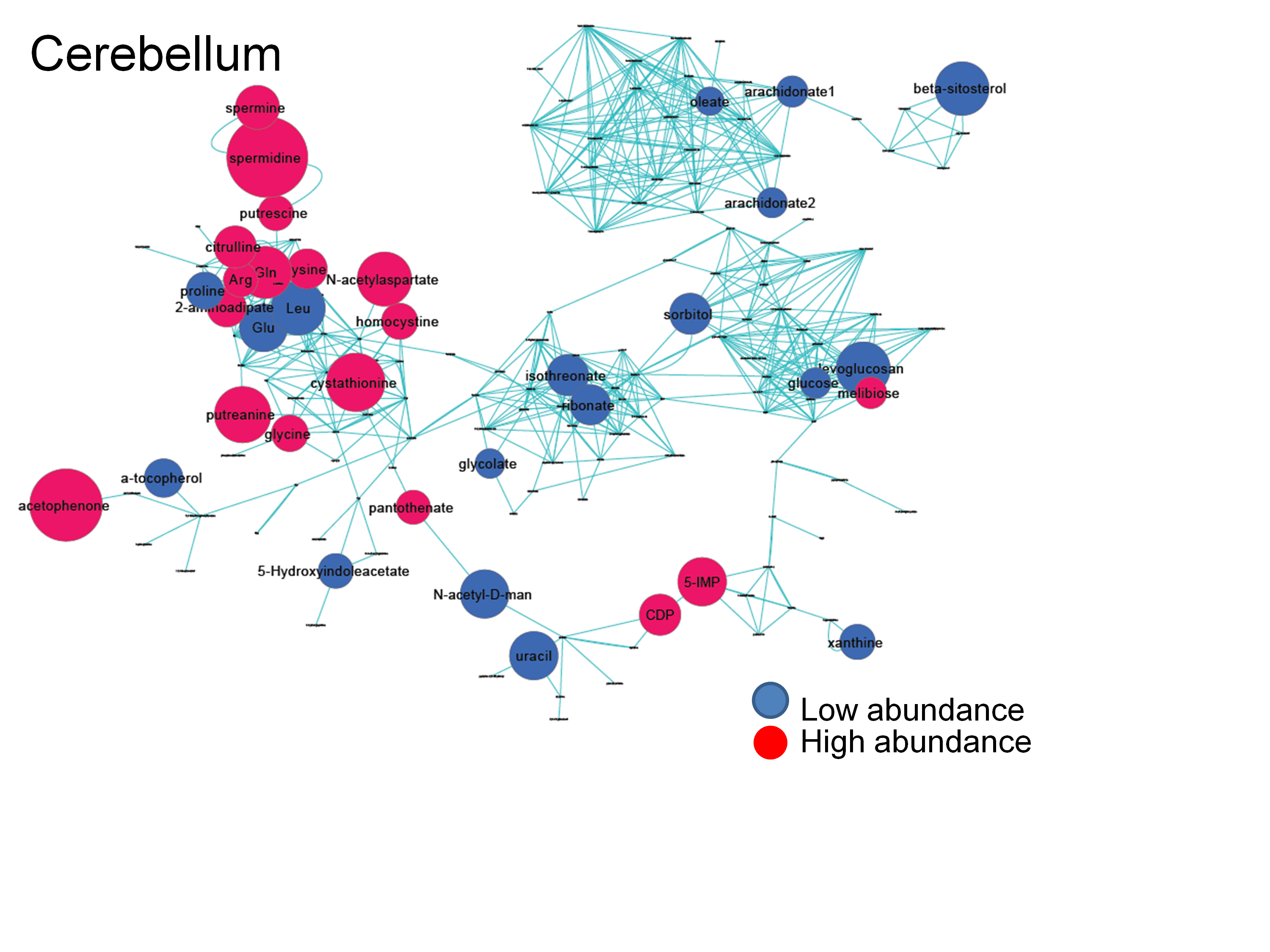

Supplement: Figure S9 — Relative pool size of primary metabolites of the cerebellum region. The metabolic network shows relative abundance of primary metabolites of the cortex compared to those of other brain regions. Blue = down regulated metabolites, red = up regulated metabolites at 0%<median false discovery rate <0.5% from SAM (n = 5 or 6). Ball sizes reflect magnitude of differential metabolite expression. Metabolites that were not significantly different were left unnamed in order to keep visual clarity. (PDF) [file pone.0068831.s009.pdf]
